# Association between Oral Hygiene Information Sources and Daily Dental and Denture Care Practices in Urban Community-Dwelling Older Adults

**DOI:** 10.3390/jcm12082881

**Published:** 2023-04-14

**Authors:** Kalliopi Konstantopoulou, Anastassia E. Kossioni

**Affiliations:** Department of Prosthodontics, School of Dentistry, National and Kapodistrian University of Athens, 11527 Athens, Greece

**Keywords:** oral hygiene, dentures, information sources, dental devices, home care, geriatric dentistry, aged, independent living

## Abstract

The purpose of this cross-sectional study was to explore the sources of daily oral hygiene information among urban community-dwelling older adults in Athens, Greece and associate them with their dental and denture care habits. One hundred and fifty-four older adults (aged 71.7 ± 9.2 years) participated in the study, and their dental status, denture use, daily oral care habits according to current gerodontology recommendations, and oral care information sources were investigated. Daily oral hygiene practices were poor, and a small number of individuals recalled having received oral hygiene advice from a dentist. Only 41.7% of the 139 dentate participants performed toothbrushing with fluoride-based toothpaste at least twice a day, and 35.9% completed regular interdental cleaning. Among 54 denture wearers, 68.5% removed their denture(s) at night, and 54% cleaned them at least twice a day. Oral hygiene information sources included dentists (for approximately half of the participants), media, friends/relatives, non-dental health care providers and dental technicians. Dentate participants who had received oral hygiene information from dentists had a greater probability of brushing their teeth with fluoride toothpaste at least twice a day (*p* = 0.049, OR = 2.15) and performing regular interdental cleaning (*p* < 0.001, OR = 29.26). Denture wearers who had received instructions about denture hygiene from dentists were more likely to use a brush and mild soap (*p* = 0.016, OR = 14.67) and remove their denture(s) at night (*p* = 0.003, OR = 8.75). Dentists should improve their oral health prevention and promotion strategies for their older patients.

## 1. Introduction

Oral health is a fundamental right of all people enabling them to enjoy a high quality of life [1]. It is an integral part of general health and a crucial element of healthy ageing [2]. Oral diseases should be addressed among other non-communicable diseases as a global public health priority [1,3], and countries should promote universal health coverage for oral health by 2030 [4]. Moreover, there is a growing interest in the association between oral and general health and the identification of oral health indicators with high prognostic or diagnostic value for general health deterioration [5,6].

One of the main causes of oral conditions, such as dental caries and periodontal disease, as well as systematic infection, is plaque accumulation due to poor oral hygiene. Periodontal disease and poor oral hygiene in teeth and dentures have been associated with aspiration pneumonia in hospitalised patients and nursing home residents [7,8]. Further, denture wearing during sleep has been associated with a 2.3-fold higher risk of the incidence of aspiration pneumonia in community-dwelling older people [9]. To reduce the number of respiratory pathogens, meticulous dental and denture hygiene and removal of dentures at night are among the recommended measures to prevent aspiration pneumonia in frail older adults [7,10]. According to the Japanese Society of Gerodontology, poor oral hygiene is one of the signs of oral hypofunction that may lead to oral frailty [11].

Three European expert reports by dentists, physicians and dental hygienists working with older people described the necessary measures to promote oral health in older people [12,13,14]. Among these measures are brushing the teeth at least twice a day with a toothbrush and fluoride toothpaste, cleaning the interdental spaces at least once a day, brushing the dentures at least twice a day with a nonabrasive denture cleanser or liquid soap combined with chemical cleansing agents, and removing the dentures during sleep.

However, although the above daily practices are easy to perform by older individuals (and their carers) and can prevent many oral and general conditions, neglected oral hygiene and poor daily oral care practices are common among older adults [3,8,15,16,17,18,19,20].

Several structural, intermediate and proximal determinants affect oral health in older adults, including poor general health, frailty, care dependency, poor oral health literacy, negative or wrong beliefs and attitudes towards oral health, lack of interest in oral health, limited professional advice, inadequate oral health policies and commercial activities/information which may manipulate their behaviour [3,4,13,19,21,22,23].

Dental patients are exposed to various sources of oral health information, including dental professionals, other healthcare workers, relatives, friends, or the media [24,25]. Young and middle-aged people in Iran received oral health information mainly from dentists and television/radio [24]. A study among Brazilian denture wearers with a mean age of 62 years has shown that 77.5% had not received any instructions on denture cleansing and 77.1% on oral care, while an association was recorded between the lack of instructions regarding oral care and the presence of denture stomatitis [26].

Poor professional support from dentists and other healthcare providers, including inadequate, inefficient or ineffective instructions on daily oral hygiene, may lead to poor daily oral care practices and various oral and systemic conditions. However, data on the sources of oral hygiene instructions among community-dwelling older people and their association with performing the recommended oral hygiene practices are scarce. Therefore, the purpose of this study was to explore the sources of daily oral hygiene and care information among urban community-dwelling older adults and associate them with their dental and denture care habits.

## 2. Materials and Methods

### 2.1. Study Design

The study had a cross-sectional design. Study participants were community-dwelling older adults who visited the dental school of the National and Kapodistrian University of Athens, Greece, for examination/treatment. The participants’ recruitment was conducted according to the following inclusion criteria: (a) being over 60 years of age, (b) being free of cognitive or sensory problems that may affect the ability to communicate with the investigators, (c) being able to speak and understand the Greek language, and (d) offering informed consent to participate. The interviews took place at the first treatment session before any discussion or demonstration of oral hygiene practices by students and faculty.

The research instruments consisted of structured oral interviews and oral clinical examinations. Oral interviews included the recording of participants’ sociodemographic characteristics, dental visitations habits, oral and denture hygiene habits, and sources of information about daily oral hygiene. Specific questions investigated the application of currently recommended oral hygiene practices for older adults, including toothbrushing at least twice a day with fluoride toothpaste, interdental brushing at least once a day, denture cleansing at least twice a day with a brush and mild soap, regular use of denture cleansing tablets and removing dentures at night. Dentate/edentulism status and the presence of removable prostheses were also recorded. The sample size was determined using the G*Power 3.1.9.4 software (www.gpower.hhu.de), which pointed at 50 participants [Chi-square test, effect size = 0.4, power of 1 − β (beta error) = 0.80, α (alpha error) = 0.05, df (degrees of freedom) = 1].

### 2.2. Statistical Analysis

Data analysis was performed anonymously. Statistical analyses included descriptive statistics and univariate analyses (Chi-square test and Fischer’s Exact test). Independent variables with statistically significant or marginally significant (*p* < 0.10) association with the recommended oral and denture hygiene practices were further included in logistic regression analyses. The dependent variables were toothbrushing at least twice a day, using regular interdental cleaning, and removal of dentures at night. The level of statistical significance was set at *p* ≤ 0.05. The analysis was performed using statistical software (IBM Corp. Released 2019. IBM SPSS Statistics for Windows, Version 26.0. Armonk, NY, USA: IBM Corp.).

## 3. Results

### 3.1. Daily Oral Hygiene Habits and Sources of Oral Care Information

A total of 154 community-dwelling older people (55 males and 99 females) with a mean age of 71.7 ± 9.2 years participated in this study. Participants’ sociodemographic characteristics are shown in Table 1. The majority belonged to the age group 60–74 years. Of the total sample, 64% were married, 27.9% were widowed, and 38.4% had attended six or fewer years of formal education. A total of 42.9% reported that they had visited the dentist within the last 12 months. Clinical examination revealed complete edentulism in 9.7% of the participants. The dental hygiene habits and sources of daily dental care information for the dentate older adults (*n* = 139) are reported in Table 2. Forty-two per cent of the respondents cleaned their teeth at least twice a day, as recommended, and 1.4% never. The most frequent method of daily dental hygiene was a manual toothbrush and fluoride toothpaste (92.1%), while interdental cleaning was performed by only 35.9% of the participants.

Only 29 (20.9%) of the dentate participants performed the recommended combination of toothbrushing at least twice a day and daily interdental cleaning. The most frequent source of oral hygiene information for toothbrushing and interdental cleaning was the dentist (49.6% and 34.5%, respectively). Other information sources included pharmacists, relatives and friends, magazines, TV, radio or internet, formal education and dental technicians. However, 35.3% and 59.0% reported that they had never obtained or did not remember to have received any information about toothbrushing or interdental cleaning, respectively.

Fifty-four participants (35.1%) used complete or partial removable dentures. Details on denture wearing, denture hygiene habits and related sources of information are presented in Table 3. The age of dentures was 11.6 ± 10.4 years (range 1–50 years). Only 29 (53.7%) of denture wearers cleaned them at least twice a day, as recommended. Mechanical brushing with toothpaste was the most frequent denture hygiene means (55.4%), and only 16.1% used a (tooth)brush and mild soap. Few participants reported brushing only with water or soda and lemon, using sodium hypochlorite or using only denture cleansing tablets. Denture cleansing tablets were used by 12 (22.2%) denture wearers, usually in combination with other methods.

Almost 70% of denture wearers removed their denture (s) at night, as strongly suggested, while 31.5% always used them (Table 3). The dentist was the most common source of denture care information (42.6%), followed by relatives and friends, pharmacists, and dental technicians. Moreover, 51.9% of denture wearers mentioned that the dentist advised them to remove their denture (s) at night. Forty-four per cent did not receive or did not remember to have received any information about daily denture hygiene and care.

Nine denture wearers (16.7%) used denture adhesives; of these participants, seven (13%) always used them, and two (3.7%) sometimes used them. The dentist was the main source of information on the use of denture adhesives (13%), followed by TV/magazines/internet (5.5%), relatives/friends (3.7%) and pharmacists (1.8%). Thirty-seven (66.1%) denture wearers did not know how often dentures should be replaced.

Twenty individuals (12.9%) used a mouth rinse on a daily basis, and 34 (22.1%) occasionally. The main source of information about mouth rinses was the dentist (16.2%), followed by media (5.8%), relatives and friends (5.2%), and pharmacists (1.3%).

### 3.2. Factors Affecting the Application of the Recommended Daily Dental Hygiene Practices among Dentate Participants

The association between toothbrushing frequency of at least twice a day among dentate older adults and several independent variables is shown in Table 4. Toothbrushing at least twice a day was statistically significantly associated with not being married (*p* = 0.046), having more years of formal education (*p* = 0.006), visiting the dentist within the past 12 months (*p* < 0.001) and having received relevant information from a dentist (0.003).

Multivariate analysis revealed statistically significant effects of education, marital status, last dental visit, and source of information on toothbrushing frequency (Table 5). Older individuals who were married (β = 0.76, OR = 2.15), those who had received at least seven years of education (β = 0.80, OR = 2.22), those who visited the dentist within the last 12 months (β = 1.04, OR = 2.84) and those informed by the dentist about daily oral hygiene (β = 0.77, OR = 2.15) were more likely to brush their teeth at least twice a day (Table 5).

Univariate analyses between regular interdental cleaning and various independent variables are presented in Table 6. More years of formal education and being informed by a dentist were statistically significantly associated with regular interdental cleaning (*p* = 0.014 and *p* < 0.001, respectively). Multivariate analysis (Table 7) demonstrated that only the source of information had a significant effect on interdental cleaning. Participants who were informed about interdental cleaning by their dentists were more likely to regularly use interdental brushes and/or dental floss (β = 3.38, OR = 29.26).

The factors affecting the combination of tooth brushing at least twice a day and regular interdental cleaning are presented in Table 8 and include more years of formal education, a dental visit within the past 12 months, and having received relevant instructions from a dentist (*p* = 0.012, *p* = 0.042 and *p* < 0.001, respectively). The multivariate analysis showed that older adults who had obtained oral hygiene information directly from the dentist had a statistically significantly higher probability of brushing their teeth at least twice per day and performing regular interdental cleaning (β = 2.40, OR = 11.03) (Table 9).

### 3.3. Factors Affecting the Application of the Recommended Daily Denture Hygiene and Care Practices

The frequency of denture hygiene was not statistically significantly associated with any of the independent parameters investigated. Source of information about denture hygiene (by a dentist) and last dental visit within the past 12 months were significantly (*p* = 0.001) and marginally significantly (*p* = 0.066) associated with denture cleaning using mechanical brushing and mild soap, while gender, age, marital status, and education did not have any significant effect. In the multivariate analysis, only the source of information remained significant (*p* = 0.016). Participants who had received instructions about denture hygiene from the dentist were more likely to use a brush and soap (β = 2.69, OR = 14.67).

Furthermore, direct information from the dentist led to an increased prevalence of denture removal at night (*p* = 0.001) (Table 10). Logistic regression has shown that those informed by the dentist about the appropriate frequency of denture use had a higher probability of removing their denture (s) at night (β = 2.17, OR = 8.75, *p* = 0.003) (Table 11).

## 4. Discussion

Under the limitations of the present study, the participants had received daily oral hygiene and care information from various sources, including dentists, media (television, magazines and the internet), formal education, relatives, friends, and other care providers, such as pharmacists and dental technicians. The oral care information obtained from dentists was associated with better adherence to the currently recommended daily oral care practices for both teeth and dentures for older adults. However, many participants reported that they had not received any kind of oral hygiene information. Moreover, for many participants, the reported dental and denture daily care habits were poor.

Almost half of the dentate participants reported that they had obtained toothbrushing instructions from their dentist but only 34.5% on interdental cleaning. Many studies have shown that the dentist is the main source of oral health information to her/his patients. As in the present study, a younger and middle-aged sample in Iran indicated that the most common oral health information source was the dentist (52.6%), followed by TV/radio, books/newspapers, family/friends and the internet, and only 1% had not obtained any relevant information [24]. Partially dentate adults in Brazil obtained information about oral hygiene mainly from their dentist or undergraduate dental students (62.2%), 5.5% from the media, and 0.8% from relatives [25]. Dental advice led to a higher possibility of having adequate oral health literacy among younger and middle-aged Brazilian immigrants in Canada [27].

Forty-three per cent of the participants reported that their dentist had educated them on denture cleaning, and 51.9% on removing the dentures at night. Lower percentages were reported by middle-aged and older individuals in Brazil (22.5%) [26] and Iran (39.6%) [28] but were higher in Turkey (90%) [29].

The findings in the present study revealed that many participants did not perform the recommended daily oral and denture hygiene practices [12,13,14]. A positive finding was the combination of a toothbrush and fluoride toothpaste (95%). Similar findings in adult and older populations have been reported in other countries [30,31]. The use of fluoride toothpaste has expanded in the past decades, particularly in developed countries. It is a major contributor to decay control and has been included in the list of essential medicines by WHO [4]. Electric toothbrushes were used by a very small number of participants (3%). Although electric toothbrushes seem to be equally or even more effective in dental plaque removal compared to conventional ones, further studies are needed to examine their applicability and affordability in older individuals [32].

Regarding toothbrushing frequency, 42% of the participants brushed their teeth at least twice a day compared to 31% in a previous study among older adults in the same geographical area [33], which is considered significant progress. However, there is large variability in relevant findings among countries [30,31,34,35].

Only 36% per cent of the participants regularly cleaned the interdental areas with dental floss and/or interdental brushes. Although there is a large variation in the frequency of interdental cleaning between studies, rates are significantly lower compared to toothbrushing [30,31]. It should be noted that only 21% of the study participants combined toothbrushing with fluoride toothpaste at least twice a day and regular interdental cleaning, and these habits have been associated with related instructions from their dentist.

Many participants (35%) used mouth rinses daily or occasionally, but only 16.2% after dental advice, as it is highly recommended to control potential side effects. Other information sources such as the media, relatives and friends and other healthcare workers played an important role in promoting this habit. A systematic review of randomised clinical trials demonstrated the wide use of mouth rinses, especially those which contain chlorhexidine, fluorides and essential oils, in the older population [36]. These findings clearly indicate that more emphasis is needed on educating non-dental healthcare providers and the public about the proper use of various mouth rinses.

Mechanical brushing with toothpaste was the most common denture hygiene practice (55.4%) despite the existing recommendations to avoid toothpaste. Previous studies among denture wearers in American, Asian and European countries also reported mechanical brushing alone (36.5–100%) or with toothpaste (29.2–88.9%) as the most prevalent denture cleaning method [26,28,34,37,38,39,40,41,42,43]. However, only 54% of the study participants cleaned their dentures at least twice a day, and only 22.2% used denture cleansing tablets as recommended [12,13,14].

Less than 70% of denture wearers removed their dentures at night, and the main contributor to this practice was previous dental advice. However, few denture wearers across the globe (23.6–58.5%) remove their dentures at night [26,28,37,38,39,40,41,44,45], although proper hygiene and nocturnal removal of dentures can reduce the incidence of aspiration pneumonia, a leading cause of death from infection among frail older persons [9,46,47,48].

Although the dentist was the main source of information about the use of denture adhesives, the media, relatives and friends also played a significant role. Denture adhesives support the quality of life of denture wearers [49], but their use should be based on professional advice [50].

Women reported better daily oral hygiene practices compared to men but not to a statistically significant level. Several previous studies in adult and older populations have associated female gender with higher level of oral health literacy and better toothbrushing and denture hygiene habits [24,41,51,52]. Participants with more than six years of education were twice as likely to brush their teeth at least twice a day. Lower educational level has been frequently reported as a significant predictor of poorer oral health literacy among middle-aged and older people [26,30,51,53,54,55,56,57]. Last dental visit was another independent predictor for increased toothbrushing frequency. However, only 45.3% of the dentate participants had visited the dentist in the past 12 months, and this percentage decreased to 39.3% in denture wearers.

Many participants did not recall having received detailed information about dental and denture care practices from any source. This score was 35.3% for toothbrushing, 59% for interdental cleaning, and 44.4% for daily denture hygiene. Patients tend to forget oral health instructions provided by their dentists [58], and this may explain both a large number of those with poor reported oral hygiene practices as well as those who reported that they had not received any information from any source. Almost one-third of a Brazilian sample of adults up to 64 years old also reported that they did not have any access to oral health information [59].

Older individuals face various degrees of declines in intrinsic capacities, such as cognitive and visual impairment, hearing loss and limited mobility [60], which may limit access to the dentist, their understanding of the provided instructions and the actual daily hygiene practices. The type of information from the dentist may have a significant effect on the level of denture cleaning [29]. Those who had obtained both written and verbal advice had the highest level of denture cleaning, followed by those who were informed only verbally or only in written [29]. It seems that the use of multiple oral health information sources may improve oral hygiene practices. Moreover, easy-to-read oral health education material enriched oral health literacy among older adults [61]. Access to digital technology may help even those with limited access to dental offices, as web-based oral health promotion programmes for older adults have been shown to improve oral health knowledge, attitudes and self-efficacy [62]. However, this requires digital literacy promotion programmes for older people.

Dental practitioners should improve their communication skills with older adults [63] and provide comprehensive oral health information adapted to individual levels of capacity. Moreover, non-dental care providers who play an important role in oral hygiene counselling of older adults and their carers should receive appropriate oral health education and training [18]. Finally, effective policies are needed to improve the oral health literacy of the public for self-care and care of others [18].

### Study Limitations

Study participants belonged to a functionally independent urban dental school sample, restricting the generalisation of the findings to functionally dependent older population groups and those living in rural areas. Also, there is a high possibility of recall bias as patients may have forgotten their dentist providing information about dental and denture hygiene. In addition, the reported oral hygiene practices, dental visitation habits and sources of oral health information may not correspond completely to reality since some participants may have adapted their answers to satisfy the investigators (Hawthorne effect). Finally, the clinical examination included only the number of natural teeth and the presence of the dentures without investigating other oral health indicators.

## 5. Conclusions

Under the limitations of the present study, the application of currently recommended daily oral hygiene practices was associated with previous relevant instructions from a dentist. However, only a small number of individuals recalled having received oral health prevention and promotion advice from a dentist. Appropriate policies should be developed and implemented to improve oral health knowledge and practices among older adults taking into consideration their specific characteristics and the heterogeneity of ageing. Dental practitioners should improve communication with their older patients with an emphasis on effective, detailed and repetitive advice. More effective and tailor-made educational strategies should be developed, including digital technology. Finally, oral health education should be provided to non-dental health care professionals who meet older adults more often than dentists.

## Figures and Tables

**Table 1:** Sociodemographic characteristics and dental visitation habits of study participants.

	*n*	%
**Sociodemographic Characteristics**		
Gender		
Male	55	35.7%
Female	99	64.3%
Total	154	100.0%
Age (years)		
<75	92	59.7%
≥75	62	40.3%
Education (years)		
≤6 years	59	38.4%
7–12 years	56	36.4%
>12 years	36	23.4%

**Table 2:** Dental hygiene habits and sources of daily dental hygiene information among dentate older individuals (*n* = 139).

	*n*	%
**Dental hygiene habits**		
**Toothbrushing frequency**		
≥Twice a day	58	41.7%
Once a day	58	41.7%
<Once a day	21	15.1%
Never	2	1.5%
**Toothbrushing means**		
Toothbrush and fluoride toothpaste	128	92.1%
Electric toothbrush and fluoride toothpaste	4	2.9%
Toothbrush and water	5	3.6%
No teeth cleaning	2	1.4%
**Interdental cleaning**		
Interdental cleaning on a regular basis	50	35.9%
Interdental brushes	30	21.6%
Dental floss	13	9.3%
Both interdental brushes and dental floss	7	5.0%
**Information source for toothbrushing**		
Dentist	69	49.6%
Pharmacist	8	5.8%
Relatives/friends	7	5%
Magazines/TV/internet	5	3.6%
Dental technician	1	0.7%
None/cannot remember	49	35.3%
**Information source for interdental cleaning**		
Dentist	48	34.5%
Pharmacist	3	2.2%
Relatives/friends	1	0.7%
Magazines/TV/internet	3	2.2%
Formal education (School/University, etc.)	1	0.7%
None/cannot remember	82	59.0%

**Table 3:** Dentures’ use, dentures’ hygiene habits and sources of information about daily dentures’ care among denture wearers (*n* = 54).

	*n*	%
**Denture use**		
Maxillary complete denture	23	42.6%
Mandibular complete denture	18	33.3%
Maxillary partial denture	21	35.2%
Mandibular partial denture	28	50.0%
**Denture hygiene frequency**		
≥Twice a day	29	53.7%
Once a day	22	40.7%
Not every day	3	5.6%
**Denture hygiene means**		
Brushing with mild soap	9	16.7%
Brushing with toothpaste	31	57.4%
Brushing only with water	6	11.1%
Brushing with soda and lemon	1	1.9%
Only rinsing with water	4	7.4%
Use of denture cleansing tablets	12	22.2%
Denture cleansing tablets only	1	1.9%
Use of sodium hypochlorite	1	1.9%
**Denture use frequency**		
During the day	30	55.6%
Always (24 h)	17	31.5%
During meals only	6	11.1%
When going out only	1	1.9%
Denture removal at night	37	68.5%
**Information source for denture hygiene**		
Dentist	23	42.6%
Pharmacist	1	1.9%
Relatives and friends	3	5.6%
Dental technician	1	1.9%
None/cannot remember	24	44.4%
**Information from the dentist for removing dentures at night**		
Yes	28	51.9%
No	26	48.1%

**Table 4:** The association of sociodemographic characteristics, dental visitation habits and source of dental hygiene information with the recommended toothbrushing frequency in dentate participants.

			Toothbrushing Frequency		
Independent Variable	*n* (%)		*n* (%)		*p*-Value
			<2 Twice/Day	≥2 Twice/Day	
Gender	Male	51 (26.7%)	29 (56.9%)	22 (43.1%)	0.797 ^a^
	Female	88 (63.3%)	52 (59.1%)	36 (40.9%)	
Age	≤74 years	89 (64%)	52 (58.4%)	37 (41.6%)	0.466 ^b^
	75–84 years	44 (31.7%)	27 (61.4%)	17 (38.6%)	
	≥85 years	6 (4.3%)	2 (33.3%)	4 (66.7%)	
Marital status	Married	90 (64.7%)	58 (64.4%)	32 (35.6%)	0.046 ^a^
	Other	49 (35.3%)	23 (46.9%)	26 (53.1%)	
Education	≤6 years	52 (37.4%)	38 (73.1%)	14 (26.9%)	0.006 ^a^
	>6 years	87 (62.6%)	43 (49.4%)	44 (50.6%)	
Last dental visit	≤12 months	63 (45.3%)	26 (41.3%)	37 (58.7%)	<0.001 ^a^
	>12 months	76 (54.7%)	55 (72.4%)	21 (27.6%)	
Source of oral health information	Dentist 73	(52.5%)	34 (46.6%)	39 (53.4%)	0.003 ^a^
	Other 66	(47.5%)	47 (71.2%)	19 (28.8%)	

^a^ Chi-square test, ^b^ Fischer’s Exact test.

**Table 5:** Multivariate analysis between toothbrushing frequency at least twice a day and independent variables.

Predictor Variables	β	SE	*p*-Value ^a^	Odds Ratio
Marital status	0.76	0.40	0.050	2.15
Education	0.80	0.41	0.050	2.22
Last dental visit	1.04	0.39	0.007	2.84
Source of oral health information	0.77	0.39	0.049	2.15

^a^ Binary Logistic Regression.

**Table 6:** The association of sociodemographic characteristics, dental visitation habits and source of daily dental hygiene information with regular interdental cleaning in dentate participants.

			Interdental Cleaning on a Regular Basis		
Independent Variable	*n* (%)		*n* (%)		*p*-Value
			No	Yes	
Gender	Male	51 (26.7%)	36 (70.6%)	15 (29.4%)	0.220 ^a^
	Female	88 (63.3%)	53 (60.2%)	35 (39.8%)	
Age	≤74 years	89 (64%)	54 (62.7%)	35 (39.3%)	0.485 ^b^
	75–84 years	44 (31.7%)	30 (68.2%)	14 (31.8%)	
	≥85 years	6 (4.3%)	5 (83.3%)	1 (16.7%)	
Marital status	Married	90 (64.7%)	56 (62.2%)	34 (37.8%)	0.548 ^a^
	Other	49 (35.3%)	33 (67.3%)	16 (32.7%)	
Education	≤6 years	52 (37.4%)	40(82.7%)	12 (17.3%)	0.014 ^a^
	>6 years	87 (62.6%)	49 (56.3%)	38 (43.7%)	
Last dental visit	≤12 months	63 (45.3%)	38 (60.3%)	25 (39.7%)	0.406 ^a^
	>12 months	76 (54.7%)	51 (67.1%)	25 (32.9%)	
Source of oral health information	Dentist	73 (52.5%)	9(18.8%)	39 (81.2%)	<0.001 ^a^
	Other	66 (47.5%)	80 (87.9%)	11 (12.1%)	

^a^ Chi-square test, ^b^ Fischer’s Exact test.

**Table 7:** Multivariate analysis between interdental cleaning and independent variables.

Predictor Variable	β	SE	*p*-Value ^a^	Odds Ratio
Education	0.52	0.51	0.327	1.67
Source of oral health information	3.38	0.49	<0.001	29.26

^a^ Binary Logistic Regression.

**Table 8:** The association of sociodemographic characteristics, dental visitation habits and source of daily dental hygiene information with toothbrushing frequency at least twice a day and interdental cleaning at least once a day.

			Toothbrushing Frequency ≥ Twice a Day and Interdental Cleaning on a Regular Basis		
Independent Variable	*n* (%)		*n* (%)		*p*-Value
			No	Yes	
Gender	Male	51 (26.7%)	41 (80.4%)	10 (19.6%)	0.782 ^a^
	Female	88 (63.3%)	69 (78.4%)	19 (21.6%)	
Age	≤74 years	89 (64%)	71 (79.8%)	18 (20.2%)	0.915 ^b^
	75–84 years	44 (31.7%)	34 (77.3%)	10 (22.7%)	
	≥85 years	6 (4.3%)	5 (83.3%)	1 (16.7%)	
Marital status	Married	90 (64.7%)	73 (81.1%)	17 (18.9%)	0.438 ^a^
	Other	(35.3%)	37 (75.5%)	12 (24.5%)	
Education	≤6 years	52 (37.4%)	47 (90.4%)	5 (9.6%)	0.012 ^a^
	>6 years	87 (62.6%)	63 (72.4%)	24 (27.6%)	
Last dental visit	≤12 months	63 (45.3%)	45 (71.4%)	18 (28.6%)	0.042 ^a^
	>12 months	76 (54.7%)	65 (85.5%)	111 (14.5%)	
Source of oral health information for teeth brushing and interdental cleaning	Dentist	73 (52.5%)	23 (18.8%)	22 (81.2%)	<0.001 ^a^
	Other	66 (47.5%)	87 (51.1%)	7 (48.9%)	

^a^ Chi-square test, ^b^ Fischer’s Exact test.

**Table 9:** Multivariate analysis between toothbrushing frequency at least twice a day combined with interdental cleaning and independent variables.

Predictor Variables	β	SE	*p*-Value ^a^	Odds Ratio
Education	0.60	0.60	0.314	1.82
Last dental visit	0.86	0.50	0.087	2.37
Source of oral health information	2.40	0.52	<0.001	11.03

^a^ Binary Logistic Regression.

**Table 10:** The association of sociodemographic characteristics, dental visitation habits and information from the dentist with denture removal at night.

			Denture Removal at Night		
Independent Variable	*n* (%)		*n* (%)		*p*-Value
			Yes	No	
Gender	Male	17 (31.5%)	11 (64.7%)	6 (35.3%)	0.683 ^a^
	Female	37 (68.5%)	26 (70.3%)	11 (29.7%)	
Age	≤74 years	23 (42.6%)	14 (60.9%)	9 (39.1%)	0.668 ^b^
	75–84 years	23 (42.6%)	17 (73.9%)	6 (26.1%)	
	≥85 years	8 (14.8%)	6 (75.0%)	2 (25.5%)	
Marital status	Married	31 (57.4%)	20 (64.5%)	11 (35.5%)	0.462 ^a^
	Other	23 (42.6%)	17 (73.9%)	6 (26.1%)	
Education	≤6 years	29 (53.7%)	22(77.4%)	7 (22.6%)	0.211 ^a^
	>6 years	25 (46.3%)	15(60%)	10 (40%)	
Last dental visit	≤12 months	22 (39.3%)	13 (59.1%)	9 (40.9%)	0.216 ^a^
	>12 months	32 (60.7%)	24 (75.0%)	8 (25.0%)	
Information from the dentist for removing dentures at night	Yes	28 (51.9%)	25 (89.3%)	3 (10.7%)	0.001 ^a^
	No	26 (48.1%)	12 (46.2%)	14 (53.8%)	

^a^ Chi-square test, ^b^ Fischer’s Exact test.

**Table 11:** The association between denture removal at night and information from the dentist.

Predictor Variable	β	SE	*p*-Value ^a^	Odds Ratio
Information from the dentist for removing dentures at night	2.17	0.73	0.003	8.75

^a^ Binary Logistic Regression.

## Data Availability

The data presented in this study are available on request from the corresponding author. The data are not publicly available due to ethical restrictions.
